# Analysis of the Constituents in “Zhu She Yong Xue Shuan Tong” by Ultra High Performance Liquid Chromatography with Quadrupole Time-of-Flight Mass Spectrometry Combined with Preparative High Performance Liquid Chromatography

**DOI:** 10.3390/molecules201119712

**Published:** 2015-11-18

**Authors:** Lin-Lin Wang, Li-Feng Han, He-Shui Yu, Mang-Mang Sang, Er-Wei Liu, Yi Zhang, Shi-Ming Fang, Tao Wang, Xiu-Mei Gao

**Affiliations:** 1 Tianjin State Key Laboratory of Modern Chinese Medicine, Tianjin University of Traditional Chinese Medicine, 312 Anshanxi Road, Nankai District, Tianjin 300193, China; lynnwlin@yeah.net (L.-L.W.); hanlifeng_1@sohu.com (L.-F.H); hs_yu08@163.com (H.-S.Y.); 1174662177sang@sina.com (M.-M.S.); liuwei628@hotmail.com (E.-W.L.); zhwwxzh@263.net (Y.Z.); fang_shiming@163.com (S.-M.F.); wangt@263.net (T.W.); 2 Tianjin Key Laboratory of TCM Chemistry and Analysis, Tianjin University of Traditional Chinese Medicine, 312 Anshanxi Road, Nankai District, Tianjin 300193, China

**Keywords:** ZSYXST, saponins, UHPLC-Q-TOF/MS

## Abstract

“Zhu She Yong Xue Shuan Tong” lyophilized powder (ZSYXST), consists of a series of saponins extracted from *Panax notoginseng*, which has been widely used in China for the treatment of strokes. In this study, an ultra-high performance liquid chromatography with quadrupole time-of-flight mass spectrometry (UHPLC-Q-TOF/MS) combined with preparative high performance liquid chromatography (PHPLC) method was developed to rapidly identify both major and minor saponins in ZSYXST. Some high content components were removed through PHPLC in order to increase the sensitivity of the trace saponins. Then, specific characteristic fragment ions in both positive and negative mode were utilized to determine the types of aglycone, saccharide, as well as the saccharide chain linkages. As a result, 94 saponins, including 20 pairs of isomers and ten new compounds, which could represent higher than 98% components in ZSYXST, were identified or tentatively identified in commercial ZSYXST samples.

## 1. Introduction

“Zhu She Yong Xue Shuan Tong” lyophilized powder, containing saponins of *Panax notoginseng*, is commonly used for treating strokes in the clinic. It can dilate blood vessels, promote blood circulation [1], and prevent thrombosis [2,3,4]. It is also reported to have a therapeutic effect on diabetes [5,6]. Its efficacy has been confirmed and is widely accepted in clinical application. The average annual sales of ZSYXST in China are about a hundred million dollars. Although it is widely used in China, its chemical constituents, especially the minor compounds, are not understood very well. Recently, there was a chemical analysis of ZSYXST by an LC/MS method, however, only 30 compounds were identified because the researchers only used a normal LC/MS method [7]. Thus, the aim of this study is to establish a comprehensive analytical method to profile the constituents of ZSYXST as much as possible.

Ultra-high performance liquid chromatography (UHPLC) is characterized by the advantages of high resolution, good sensitivity, high speed of analysis and high peak capacity [8]. Quadrupole time of flight mass spectrometry (Q-TOF/MS) has already been widely used for structural characterization of unknown saponins [9,10,11]. Thus, combining UHPLC with Q-TOF/MS (UHPLC-Q-TOF/MS) could be an effective method to identify the chemical constituents in ZSYXST. There are four major saponins (notoginsenoside R_1_, ginsenosides Rg_1_, Re, and Rb_1_) which together represent more than 85% in ZSYXST, and these four major saponins could significantly decrease the sensitivity of minor saponins in the LC-MS fingerprint. Preparative high performance liquid chromatography (PHPLC) is a separation and purification technology with the advantages of high separation efficiency, sensitive detection and automated collection of fractions. This study utilized PHPLC to remove the major ingredients from ZSYXST samples in order to decrease the influence of the major saponins on the MS detection of the minor saponins.

In this paper, the structural characteristics of saponins from ZSYXST were investigated and illuminated using UHPLC-Q-TOF/MS and a target MS/MS data acquisition strategy. The full MS scan provided protonated or deprotonated molecules in their intact form, while the target MS/MS scan provided fragment information. The fragmentation patterns of the reference saponins were investigated first, and the types of aglycone, sequences and linkage positions of saccharide chains could be deduced accurately according to some diagnostic fragments pathways in both positive and negative modes. Finally, with the help of fragmentation pathway rules and finding compounds by the molecule feature in the Agilent Mass Hunter Workstation Software (Version B.02.00), 94 saponins, including 20 pairs of isomers, which could represent more than 98% of the components were identified or tentatively identified in commercial ZSYXST samples. Based on the literature and the SciFinder database, compounds **1**, **2**, **18**, **20**, **21**, **22**, **50**, **51**, **59**, **90** were speculated to be new saponins in ZSYXST.

## 2. Results and Discussion

### 2.1. Optimization of MS Conditions

In order to obtain better MS response, cone voltage and CE were optimized. According to our research and literature data [12,13,14,15], the cone voltage was set to 120 V and 175 V in positive and negative mode, respectively. CE was dynamically adjusted from 45 to 70 V according to the *m/z* of precursor ions in the negative MS/MS mode, such as 45 V for *m/z* 650–750, 50 V for *m/z* 750–850, 55 V for *m/z* 850–950, 60 V for *m/z* 950–1050, 65 or 70 V for *m/z* > 1050.

### 2.2. MS Cleavage Rules of Saponins in ZSYXST

According to cleavage pathway of reference compounds and literatures, some diagnostic rules for the identification of compounds in ZSYXST can be summarized.

#### 2.2.1. Differentiation and Classification of Diverse Saponins in ZSYXST

**Figure 1:**
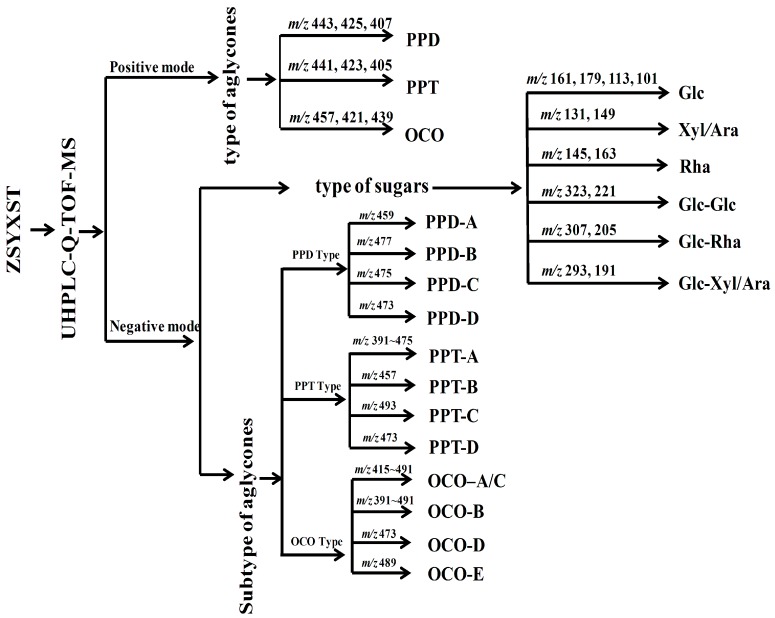
A diagram for rapid classification and identification of saponins by characteristic ions in MS and MS/MS mode.

There were three main types of saponins in ZSYXST (Figure 1), namely protopanaxadiol (PPD), protopanaxatriol (PPT), and ocotillol (OCO) types [16,17]. According to the literature [12,15], there are some fragmentation rules for the sugar chains of saponins in *P. notoginseng.* The sugar moieties of PPD type are usually attached at the C-3 or C-3, 20 positions, while PPT sugars are attached at C-6 or C-6, C-20. OCO type usually form glycosides at the C-6 position when there as a five membered epoxy ring at C-20 [18,19,20]. According to [12,21] and our results, some characteristic ions could be used for deducing aglycone types, for example, PPD-type could be identified by diagnostic fragment ions at *m/z* 443, 425 and 407, PPT-type by *m/z* 441, 423 and 405, and OCO-type by *m/z* 457, 439 and 421 (Figure 1).

#### 2.2.2. Differentiation of Sub-Types of Aglycone

**Figure 2:**
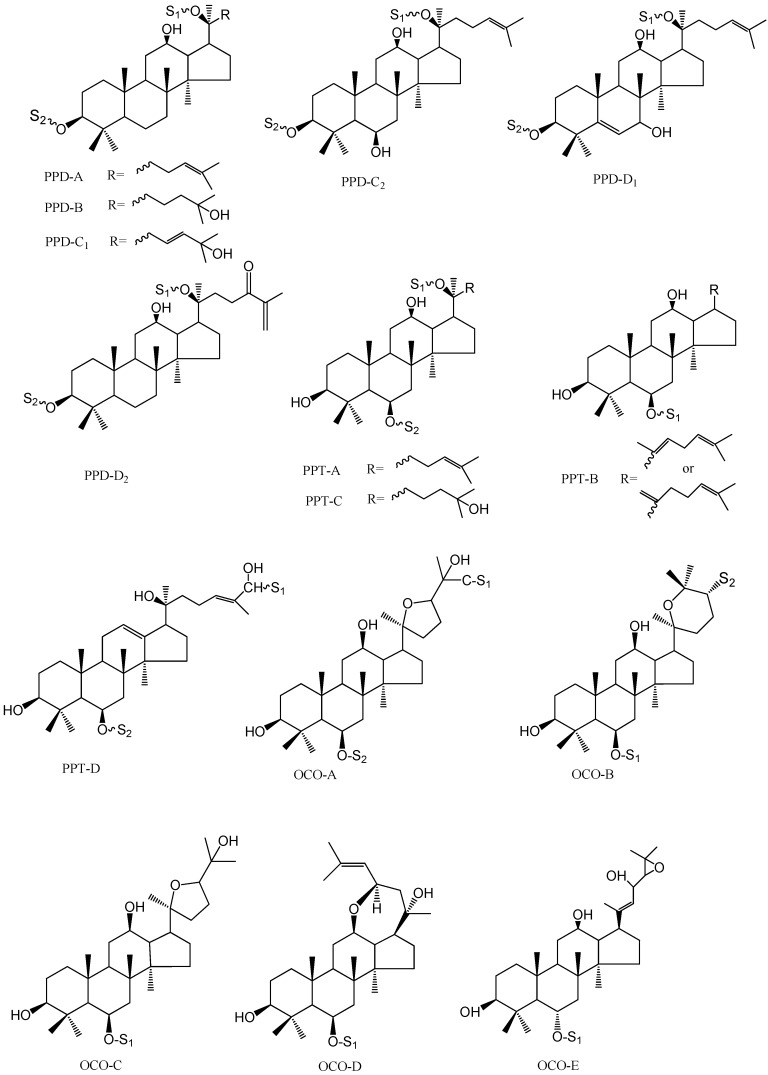
The structures of different types of saponin aglycone.

With the help of positive MS spectra data, the aglycone types could be deduced. However, there were several aglycone sub-types. Fortunately, those sub-types could be distinguished by characteristic fragment ions between *m/z* 390 to 500 in the negative MS/MS spectra. Specifically, PPD-A type could be observed at *m/z* 459, PPD-B type at *m/z* 477, PPD-C type at *m/z* 475, and PPD-D type at *m/z* 457, while the ions at *m/z* 391–475 could be attributed to PPT-A type, *m/z* 457 to PPT-B type, *m/z* 493 to PPT-C type, *m/z* 473 to PPT-D type; in OCO types, the ions at *m/z* 415–491 belonged to OCO-A or -C type, however, a neutral loss of 180 Da from precursor ions, it could be attributed to OCO-A type, as for OCO-B, -D, -E types, diagnostic ions at *m/z* 391–491, 473, and 489 could be observed, respectively (Figure 1 and Figure 2).

#### 2.2.3. Differentiation of Sugar Moieties

In negative MS/MS spectra, it could be found that the terminal sugar moiety exposed to the outside in the spatial structure would cleave first. For example, in PPD type, the end sugar moiety at C-3 was cleaved first, and then the terminal sugar moiety at C-20 would cleave afterwards. However, in the PPT type, the terminal sugar moiety at C-20 was eliminated first, and then the end sugar moiety at C-6 would cleave later. OCO type saponins also conformed to this rule.

Sugar moieties linked to an aglycone through glycoside ether bonds at C-3, C-6 and C-20 or other positions, cleaved successively from the saponins. The most common sugar neutral losses were 132 Da, 146 Da, 162 Da, 278 Da, 294 Da and 324 Da, which correspond to arabinose/xylose (Ara/Xyl), rhamnose (Rha), glucose (Glc), Rha-Ara/Xyl, Glc-Ara/Xyl and Glc-Glc, respectively. In the lower mass region of negative MS/MS spectra, characteristic fragment ions could be observed to further identify sugar moieties, such as *m/z* 161, 179, 119, 113, 101 for Glc, *m/z* 145, 163 for Rha, *m/z* 131, 149 for Xyl or Ara, *m/z* 323 or 221 for Glc-Glc, *m/z* 307, 205 for Glc-Rha, and *m/z* 293,191 for Glc-Xyl/Ara (Figure 1).

In addition, in the lower mass region of positive MS spectra, characteristic fragment ions also could be observed to further authenticate sugar moieties, such as *m/z* 325 for Glc-Glc, *m/z* 309 for Glc-Rha, *m/z* 295 for Glc-Xyl/Ara (Table 1).

**Table 1:** The characteristic ions of saponins in ZSYXST.

Comp.	[M − H]^−^	[M + COOH]^−^	[M − H]^−^/[M + COOH]^−^	Diagnostic Ions of Sugar Moieties in Negative Mode	Diagnostic Ions of Aglycone Types in Positive Mode
**1**	965.5198	1011.5278	0.1	131.0312, 191.0668	439.3453, 457.3560
**2**	965.5237	1011.5291	0.15	131.0310, 191.1745	439.3459, 457.3559
**3**	979.5387	1025.5451	0.2	205.0648	421,1231, 439.1502
**4**	979.5407	1025.5464	0.2	145.0321, 205.0667	439.3480, 457.3572
**5**	801.4635	847.4662	0.1	221.0687	407.3257, 425.3360
**6**	815.4807	861.4881	0.14	221.0117	421.3436, 439.3547
**7**	947.5170	993.5230	0.18	131.0287, 191.0432, 221.0223	421.3586, 457.3660
**8**	815.4807	861.4881	0.14	221.0654	421.3446, 439.3581
**9**	815.4669	861.4817	0.02	323.0378	421.3324, 439.3512, 457.2314
**10**	815.4814	861.4820	0.09	323.0665	421.3477, 439.3594, 457.3701
**11**	815.4701	861.4836	0.1	323.0656	421.3577, 439.3659, 457.3730
**12**	817.4872	863.4982	0.8	323.2112	423.3736, 441.3848
**13**	799.4312	845.4598	20	221.0599	405.3408, 423.3536, 441.3938
**14**	961.5332	1007.5382	0.1	145.0595, 205.0690	421.3660, 439.3758, 457.3886
**15**	961.5328	1007.5333	0.15	205.4532	421.3748, 439.3861, 457.3971
**16**	815.4673	861.4815	0.1	323.0454	439.3907, 457.4193
**17**	815.4668	861.4817	0.15	323.0452	421.3840, 439.3967, 457.4080
**18**	813.4677	859.4739	0.1		421.3640, 439.3767, 457.4070
**19**	961.5323	1007.5366	0.2	205.9385	421.3975, 439.4145, 457.4258
**20**	959.5194	1005.5220	0.25	221.0431	421.3841, 439.3967, 457.4081
**21**	813.4591	859.4652	0.1		421.4140, 439.4235, 457.4046
**22**	959.5158	1005.5205	0.3	221.0446	421.3880, 439.4228
**23**	1093.5734	1139.5771	40	221.0995, 323.0662	405.4047, 423.4172, 441.4303, 325.1562, 295.1434
**24**	1093.5719	1139.5767	14	221.0676	405.3985, 423.4120, 441.4245, 325.1519, 295.1366
**25**	961.5409	1007.5460	1.5	221.0670	405.3973, 423.4204, 441.4223, 325.1495
**26**	1093.5705	1139.5761	10	191.0538, 221.0668	405.3913, 423.4036, 441.3970
**27**	961.5305	1007.5341	0. 1	221.0597	405.3926, 423.4051, 441.4172
**28**	1107.5870	1153.5909	0.5	205.0700, 221.0662	405.3926, 423.4051, 441.4132
**29**	961.5303	1007.5361	0.8	221.0663	405.3910, 423.4035, 441.4157, 325.1456
**30**	1107.5789	1153.5908	0.5	205.0684, 221.0661	405.3886, 423.4016, 441.4128, 309.1526
**31**	961.5294	1007.5278	0.3	221.0662	405.3874, 423.4002, 441.4121
**32**	961.5289	1007.5335	0.3	221.0650	405.3848, 423.3977, 441.4089
**33**	931.5210	977.5270	0.2	131.0349, 191.0570	405.3806, 423.3936, 441.4050
**34**	961.5297	1007.5350	0.4	221.0661	405.3794, 423.3909, 441.4028
**35**	961.5295	1007.5324	0.4	221.0663	405.3898, 423.3911, 441.4001
**36**	931.5179	977.5240	0.1	191.0561, 221.0598	405.3765, 423.3878, 441.3995
**37**	961.5277	1007.5328	0.75	221.0660	405.3738, 423.3857, 441.3973
**38**	1121.5639	1167.5655	10	221.0599	407.3630, 425.3740, 443.3849
**39**	959.1231	1005.1253	0.35	221.0664	407.3597, 425.3721, 443.3820
**40**	799.4673	845.4847	0.1		405.3730, 423.3859, 441.3968
**41**	945.5324	991.5386	0.2	145.0346, 205.1696	405.3655, 423.3810, 441.3920, 309.1347
**42**	1255.6201	1301.6254	10	131.0295, 191.0599, 221.0661	405.3766, 423.3890, 441.3995, 295.1264, 325.1313
**43**	1141.5875	1187.5921	3	221.0661	423.3764, 441.3898
**44**	979.5352	1025.5410	100	221.0665	423.3764, 441.3898
**45**	1123.5721	1169.5763	5	221.0660, 323.0961	405.3629, 423.3588
**46**	901.5177	947.5063	0.88	191.0660	405.3629, 423.3588, 441.3587
**47**	769.3977	815.4032	0.15	131.0231, 191.0623	405.3629, 423.3588
**48**	901.5008	947.5050	0.8	191.0601	405.3629, 423.3588, 441.3887
**49**	769.4713	815.4661	0.2	191.0600	405.3587, 423.3712, 441.3816
**50**	959.5032	1005.5082	0.4	221.0672	421.3554, 439.3660, 457.3768
**51**	1109.5496	1155.5567	2.5	221.0670	421.3541, 439.3654
**52**	769.4673	815.4610	0.15	191.0643	405.3584, 423.3694
**53**	961.5108	1007.5159	0.4	221.0667	405.3558 , 423.3683, 441.3798
**54**	1123.5610	1169.5643	2.5	221.0661	405.3558 , 423.3683, 441.3798
**55**	769.4412	815.4537	0.1	131.0329	405.3565, 423.3679, 441.3781
**56**	915.5043	961.5123	60	131.331, 205.0690	405.3565, 423.3679, 441.3781
**57**	1123.5456	1169.5495	4	221.0653	423.3781, 441.3878, 325.1177
**58**	1123.5427	1169.5475	4	221.0644, 323.0924	405.3552, 423.3661, 441.3768
**59**	1121.5226	1167.5242	5	221.0655, 323.0970	421.3487, 439.3613, 457.3717, 325.1176
**60**	1125.5476	1171.5509	4	221.0657, 323.0960	407.3671, 425.3739
**61**	1123.5306	1169.5326	4	221.0651	405.3552, 423.3649, 441.3748
**62**	797.4781	843.4276	0.1		421.3488, 439.3595, 457.3695
**63**	1123.5154	1169.5164	3	221.0645	405.3554, 423.3641, 441.3708
**64**	947.4713	993.4633	0.25	191.0598, 221.0661	421.3447, 439.3612, 457.3701
**65**	961.4723	1007.4753	0.3	221.0644	407.3661, 425.3773
**66**	781.4321	827.42225	0.1		405.3531, 423.3637, 441.3738
**67**	961.4687	1007.4704	0.2	221.0660	405.3523, 423.3623, 441.3754
**68**	961.4623	1007.4634	6	221.0664	405.3534, 423.3665, 441.3771
**69**	799.4215	845.4232	0.3	221.0612	405.3503, 423.3621, 441.3719
**70**	961.4620	1007.4617	0.2	221.0618	405.1917, 423.3524, 441.3701
**71**	961.4630	1007.4635	0.7	221.0660	405.3501, 423.3633, 441.3721
**72**	769.4185	815.4207	0.35	131.0304, 191.0624	405.3499, 423.3609, 441.3706
**73**	799.4331	845.4304	0.8	221.0588, 323.0889	405.3489, 423.3597, 441.3700, 325.1119
**74**	959.4537	1005.4554	0.22	221.0580	439.3546, 457.3632, 325.1057
**75**	1371.5895	1417.5705	100	131.0298, 221.0645, 293.0659, 323.0926	407.3640, 425.3740, 443.3839, 325.1109, 295.1006
**76**	901.4601	947.4621	0.8	191.0599	407.3629, 4253724, 443.3830
**77**	901.4614	947.4655	0.6	131.0334, 191.0621	407.3621, 4253733, 443.3843
**78**	783.4448	829.4493	0.1	145.0247, 205.0579	405.3472, 423.3587, 441.3686
**79**	1239.5732	1285.5715	15	221.0642, 293.0883, 323.1087	407.3631, 425.3733, 443.3838, 325.1097, 295.0995
**80**	637.3796	683.4054	0.1		405.3461, 423.3562, 441.3665
**81**	1269.5894	1315.5809	50	221.0468, 323.0734	407.3614, 425.3722, 443.3799
**82**	1239.5892	1315.5879	85	131.0342, 191.0660, 221.0661, 323.0979	407.3624, 425.3726, 443.3827
**83**	1105.5357	1151.5401	0.1	221.0664	405.3459, 423.3565, 441.3661
**84**	1105.5425	1151.5449	0.1	221.0596	405.3421, 423.3554, 441.3667
**85**	1239.2331	1285.2377	40	131.0121, 191.0366, 221.0663	407.3665, 425.3779, 443.3760
**86**	1107.5643	1153.5662	10	221.0488, 323.0729	407.3613, 425.3719, 443.3813
**87**	1107.5632	1153.5643	6.6	221.0599	407.3601, 425.3677, 443.3805
**88**	1077.5696	1123.5746	3.3	131.0257, 191.0466, 221.0540	407.3583, 425.3682, 443.3781
**89**	1077.5705	1123.5761	3.3	131.0265, 191.0453, 221.0536	407.3583, 425.3690, 443.3800
**90**	943.5121	989.5183	8	221.0662	405.3433, 423.3545, 441.3794
**91**	945.5351	991.5398	0.3	221.5863	407.3622, 425.3730, 443.3826, 325.1087
**92**	945.5458	991.5516	0.35	221.0664, 323.0721	407.3829, 425.3941, 443.4048, 325.1278
**93**	619.4279	665.4239	0.1		405.4064, 423.4219, 441.4347
**94**	619.4255	665.4245	0.1		405.4064, 423.4202, 441.4332

#### 2.2.4. Identification of Sugar Chains at C-20 by the [M − H]^−^ to [M + COOH]^−^ Peak Ratio

**Figure 3:**
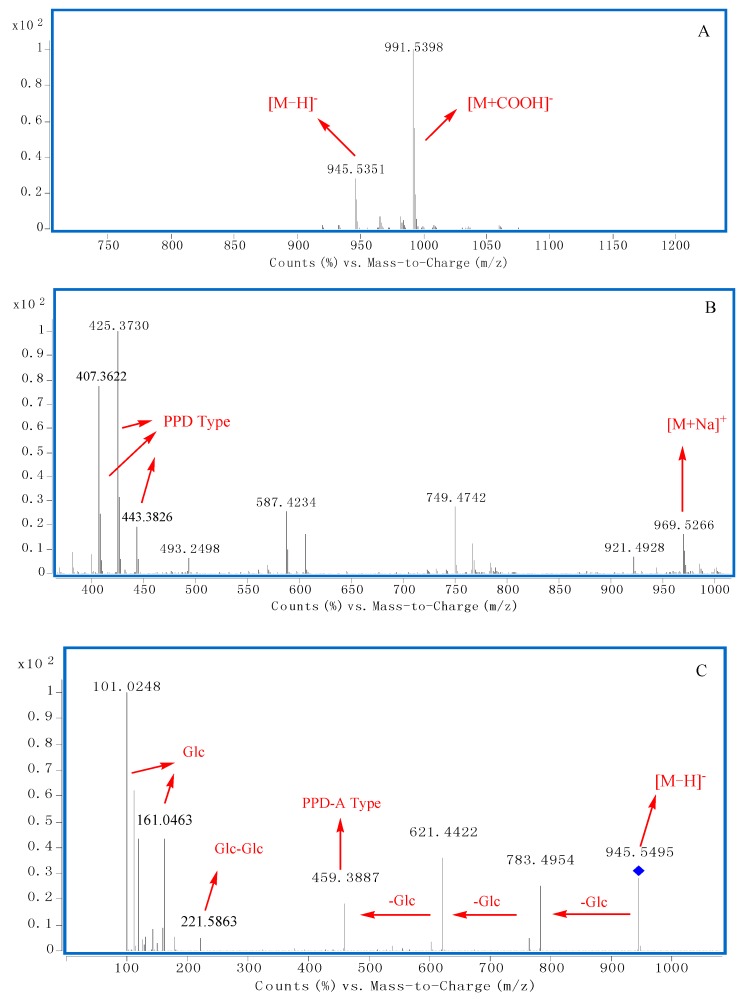
The typical TOF-MS spectra and fragmentation pathways of compound **91**. (**A**) MS spectrum in negative mode; (**B**) MS spectrum in positive mode; (**C**) MS/MS spectrum in negative mode.

**Figure 4:**
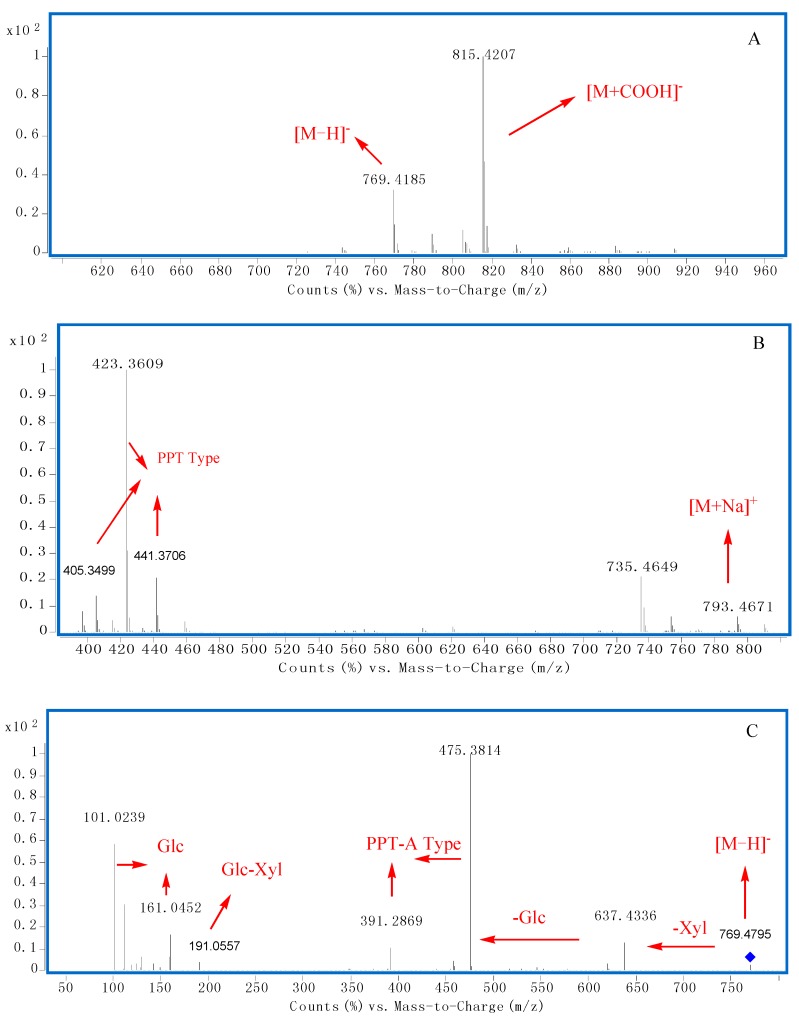
The typical TOF-MS spectra and fragmentation pathways of compound **72**. (**A**) MS spectrum in negative mode; (**B**) MS spectrum in positive mode; (**C**) MS/MS spectrum in negative mode.

**Figure 5:**
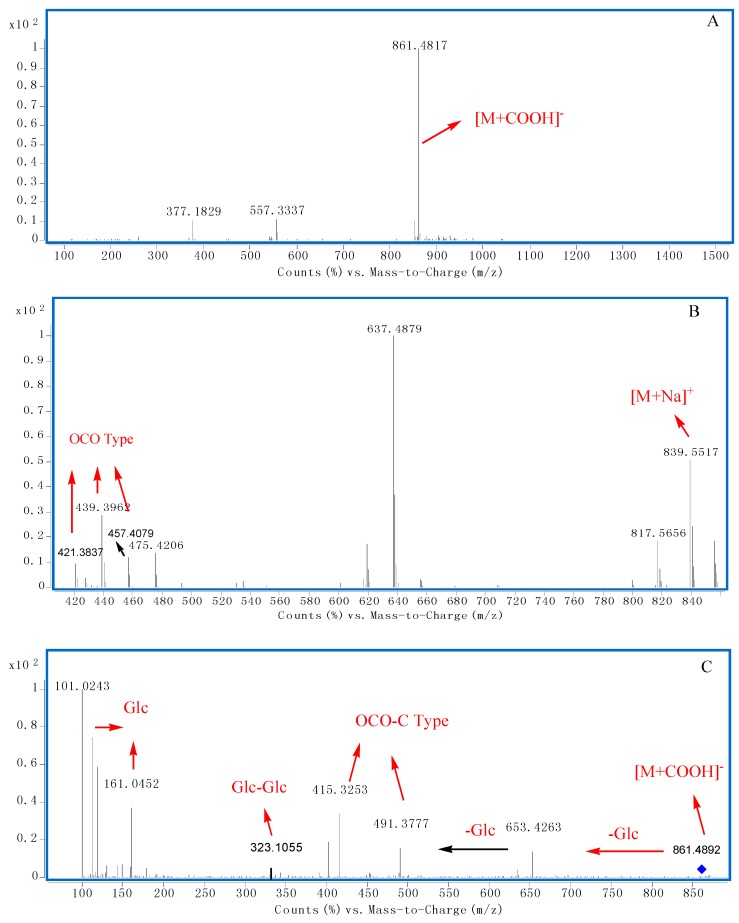
The typical TOF-MS spectra and fragmentation pathways of compound **9**. (**A**) MS spectrum in negative mode; (**B**) MS spectrum in positive mode; (**C**) MS/MS spectrum in negative mode.

There was an interesting phenomenon in the negative MS spectra. The ratio of quasi−molecular ions {[M − H]^−^ to [M + COOH]^−^} were related to the sugar chains at C-20. When there were more than one sugar located at C-20, the peak ratio of [M − H]^−^ to [M + COOH]^−^ was higher than 0.5, however, when there was only one sugar or none linked at C-20, the peak ratio would be lower than 0.5 (Figure 3A, Figure 4A and Figure 5A). These characteristics could be explained by the existence of the space effect.

### 2.3. Identification of Compounds in ZSYXST

#### 2.3.1. Identification of PPD Type Saponins

In Figure 3A,B, the molecular weight of compound **91** could be deduced as 946 through the quasi−molecular ions at *m/z* 945.5351 [M − H]^−^, 991.5398 [M + COOH]^−^ and 969.5266 [M + Na]^+^. The peak ratio of [M − H]^−^ to [M + COOH]^−^ was about 0.3, which indicated that the number of sugar moities at C-20 was less than one (Figure 3A). Furthermore, diagnostic ions at *m/z* 407, 443, 425 could be observed which indicated that the saponin should be PPD Type (Figure 1 and Figure 3B). In the MS/MS spectrum of [M − H]^−^ (Figure 3C), fragment ions at *m/z* 783.4954, 621.4422 and 459.3887 could be deduced as three successive neutral losses of glucose. 

From the diagnostic ions at *m/z* 459, the aglycone could be deduced to be PPD-A type (Figure 1 and Figure 2). Fragment ions at *m/z* 221 indicated there was a Glc-Glc chain linked to the aglycone. According to the above diagnostic ions and comparison of the retention time with a reference, **91** could unambiguously be identified as ginsenoside Rd. Similar fragment ions were observed in **92**.

Compound **75** produced [M − H]^−^ ions at *m/z* 1371.5895 and the peak ratio of [M − H]^−^ to [M + COOH]^−^ was 100 in negative mode. In MS/MS mode, diagnostic fragment ions of sugar neutral loss at *m/z* 1107.5948 [M − H − 132 − 132]^−^, 945.5704 [M − H − 132 − 132 − 162]^−^, 783.4893 [M − H − 132 − 132 − 162 − 162]^−^, 459.5118 [M − H − 132 − 132 − 162 − 162 − 162 − 162]^−^ and Glc-Glc diagnostic fragment ions at *m/z* 221.0645, 293.0659, 323.0926 were observed. In positive mode, *m/z* 407.3640, 425.3740, 443.3839 indicate **75** belongs to the PPD type. According to the above MS cleavage rules and the literature [17], compound **75** was easily identified as notoginsenoside D. 

Compound **88** generated [M − H]^−^ and [M + COOH]^−^ ions at *m/z* 1077.5696 and 1123.5746, the peak ratio of [M − H]^−^ to [M + COOH]^−^ was 3.3 in negative mode. PPD Type diagnostic ions at *m/z* 407.3583, 425.3682, 443.3781 were obtained in positive mode. According to negative MS/MS diagnostic fragment ions, such as *m/z* 945.4812 [M − H − 132]^−^, 783.4405 [M − H − 132 − 162]^−^, 621.4014 [M − H − 132 − 162 − 162]^−^, 459.3421 [M − H − 132 − 162 − 162 − 162]^−^, as well as literature data [12], **88** was identified as notoginsenoside L. Similar fragment ions were observed in compound **89**.

In the positive MS spectrum, compound **60** produced ions at *m/z* 407.3671, 425.3739 which could be used to identify it as PPD type. In the negative MS spectrum, the peak ratio of [M − H]^−^ (*m/z* 1125.5476) and [M + COOH]^−^ (*m/z* 1171.5509) was about 4, which indicated that there was more than one sugar linked at C-20. In the MS/MS spectrum, characteristic fragment ions at *m/z* 963.5411 [M − H − 162]^−^, 801.4900 [M − H − 162 − 162]^−^, 635.4389 [M − H − 162 − 162 − 162]^−^, 477.3889 [M − H − 162 − 162 − 162 − 162]^−^, Glc-Glc diagnostic fragment ions at *m/z* 221.0657, 323.0960, and PPD-B type characteristic fragment ions at *m/z* 477.3889 were all observed. According to the MS fragment rules, **60** was tentatively identified as PPD-B-S_1_(Glc-Glc)-S_2_(Glc-Glc).

Similarly, with the help of MS cleavage rules, reference compounds and literature data, another eight PPD type saponins were identified. Thus compounds **5**, **38**, **39**, **79**, **81**, **82**, **85**, **86**, **87** were identified or tentatively identified as ginsenoside C-Y_1_, quinquenoside IV, notoginsenoside G, chikusetsusaponin VI, ginsenoside Ra_0_/quinquenoside V, notoginsenoside Fa, ginsenoside Ra_3_, ginsenoside Rb_1_, isomer of ginsenoside Rb_1_, respectively (Table 1 and Table 2).

#### 2.3.2. Identification of PPT Type Saponins

According to the quasi−molecular ions at *m/z* 769.4185 [M − H]^−^, 815.4207 [M + COOH]^−^ and 793.4671 [M + Na] ^+^ , the molecular weight of compound **72** should be 770 (Figure 4A,B). The peak ratio of [M − H]^−^ to [M + COOH]^−^ was about 0.35, which indicated that there was less than one sugar linked at C-20 (Figure 4A). Furthermore, the aglycone type could be identified as PPT Type through diagnostic ions at *m/z* 405, 441, 423 (Figure 1 and Figure 4B). In the MS/MS spectrum of [M − H]^−^ (Figure 4C), fragment ions at *m/z* 637.4336, 475.3814 could be deduced to represent neutral losses of xylose and glucose successively or simultaneously. Fragment ions at *m/z* 191 indicated there was a Glc-Xyl chain in **72**. Characteristic ions at *m/z* 391 and 475 indicated the aglycone should be PPT-A type (Figure 1 and Figure 4C). According to the peak ratio rule of [M − H]^−^ to [M + COOH]^−^, we could deduce that the Glc-Xyl chain should be linked at the C-6 position of the aglycone. As a result, compound **72** could be identified as notoginsenoside R_2_. 

Compound **23** produced [M − H]^−^ ions at *m/z* 1093.5734 in negative mode, while the peak ratio of [M − H]^−^ to [M + COOH]^−^ was 40. In MS/MS mode, characteristic fragment ions at *m/z* 961.5389 [M − H − 132]^−^, 637.4333 [M − H − 132 − 162 − 162]^−^, 475.3832 [M − H − 132 − 162 − 162 − 162]^−^, 391.2772 and Glc-Glc diagnostic fragment ions at *m/z* 221.0995, 323.0662 were obtained. In positive mode, it produced ions at *m/z* 405.4047, 423.4172, 441.4303 which could be used to identify it as PPT-A type. According to the retention time and MS fragment rules, **23** was unambiguously identified as sanchirhinoside A_6_. Similar diagnostic fragment ions were observed in compounds **24** and **26**.

**Table 2:** Identification of compounds in ZSYXST.

Comp.	Rt (min)		MS/MS *m/z*	Area%	Identification
**1**	7.54	965.5198	785.9465 [M − H − 180]^−^, 653.3969 [M − H − 180 − 132]^−^, 491.3843 [M − H − 180 − 132 − 162]^−^, 415.3350	0.019	OCO-A-S_1_(Glc)-S_2_(Glc-Xyl/Ara) *
**2**	8.611	965.5237	785.9489 [M − H − 180]^−^, 653.3794 [M − H − 180 − 132]^−^, 491.3654 [M − H − 180 − 132 − 162]^−^, 415.3229	0.006	isomer of OCO-A-S_1_(Glc)-S_2_(Glc-Xyl/Ara) *
**3**	8.811	979.5387	799.4633 [M − H − 180]^−^, 635.3669 [M − H − 180 − 146]^−^, 491.3370 [M − H − 180 − 146 − 162]^−^, 415.3301	0.016	OCO-A-S_1_(Glc)-S_2_(Glc-Rha) [14]
**4**	10.189	979.5407	799.4532 [M − H − 180]^−^, 635.3868 [M − H − 180 − 146]^−^, 491.3569 [M − H − 180 − 146 − 162]^−^, 415.3277	0.006	isomer of OCO-A-S_1_(Glc)-S_2_(Glc-Rha) [14]
**5**	12.992	801.4635	639.4097 [M − H − 162]^−^, 477.3605 [M − H − 162 − 162]^−^	0.006	ginsenoside C-Y_1_ [22]
**6**	13.699	815.4807	653.4212 [M − H − 162]^−^, 491.3770 [M − H − 162 − 162]^−^, 391.2872	0.032	OCO-B-S_1_(Glc-Glc)-S_2_(OH) [23]
**7**	13.852	947.5170	785.4339 [M − H − 162]^−^, 623.4062 [M − H − 162 − 162]^−^, 491.4131 [M − H − 162 − 162 − 132]^−^, 415.3310	0.032	vinaginsenoside R_5_ or yesanchinoside C [24,25]
**8**	14.151	815.4801	653.4276 [M − H − 162]^−^, 491.3788 [M − H − 162 − 162]^−^, 391.2878	0.032	isomer of OCO-B-S_1_(Glc-Glc)-S_2_(OH)
**9**	14.252	861.4892	653.4263 [M − H − 162]^−^, 491.3777 [M − H − 162 − 162]^−^, 415.3253	0.010	majonoside R_1_ [12,19]
**10**	14.582	815.4814	653.4162 [M − H − 162]^−^, 491.3716 [M − H − 162 − 162]^−^, 415.3533	0.019	isomer of majonoside R_1_ [12,19]
**11**	15.03	815.4701	653.4162 [M − H − 162]^−^, 491.3716 [M − H − 162 − 162]^−^, 415.3519	0.151	isomer of majonoside R_1_ [12,19]
**12**	15.948	817.4872	655.4462 [M − H − 162]^−^,493.3934 [M − H − 162 − 162]^−^	0.048	PPT-C-S_1_(Glc-Glc)-S_2_(H) [26]
**13**	16.431	799.4312	799.4678 [M − H − 162]^−^, 637.4793 [M − H − 162 − 162]^−^, 475.3866 [M − H − 162 − 162 − 162]^−^, 391.2904	0.013	ginsenoside Rf [27]
**14**	16.714	961.5332	799.4679 [M − H − 162]^−^, 653.4002 [M − H − 162 − 146]^−^, 491.3304 [M − H − 162 − 146 − 162]^−^, 391.2772	0.032	OCO-B-S_1_(Glc-Rha-Glc)-S_2_(CH_3_) [26]
**15**	17.597	961.5328	799.5297 [M − H − 162]^−^, 653.4002 [M − H − 162 − 146]^−^, 491.3693 [M − H − 162 − 146 − 162]^−^, 391.8943	0.032	isomer of OCO-B-S_1_(Glc-Rha-Glc)-S_2_(CH_3_)
**16**	18.398	815.4868	653.4263 [M − H − 162]^−^, 491.3777 [M − H − 162 − 162]^−^, 415.3361	0.515	isomer of majonoside R_1_ [12,19]
**17**	18.092	815.4673	653.4373 [M − H − 162]^−^, 491.3788 [M − H − 162 − 162]^−^, 415.3415	0.032	isomer of majonoside R_1_ [12,19]
**18**	19.481	859.4723	651.4111 [M − H − 162]^−^, 489.3579 [M − H − 162 − 162]^−^	0.019	OCO-E-S_1_(Glc-Glc) *
**19**	20.247	961.5323	799.4824 [M − H − 162]^−^, 653.4002 [M − H − 162 − 146]^−^, 491.3558 [M − H − 162 − 146 − 162]^−^, 391.3441	0.016	isomer of OCO-B-S_1_(Glc-Rha-Glc)-S_2_(CH_3_) [21]
**20**	21.142	959.5194	797.4207 [M − H − 162]^−^, 635.3567 [M − H − 162 − 162]^−^, 473.3460 [M − H − 162 − 162 − 162]^−^	0.016	OCO-D-S_1_(Glc-Glc-Glc) *
**21**	22.39	859.4721	651.4113 [M − H − 162]^−^, 489.3635 [M − H − 162 − 162]^−^	0.023	isomer of OCO-E-S_1_(Glc-Glc) *
**22**	23.909	959.5158	797.4575 [M − H − 162]^−^, 635.4076 [M − H − 162 − 162]^−^, 473.3611 [M − H − 162 − 162 − 162]^−^	0.016	isomer of OCO-D-S_1_(Glc-Glc-Glc) *
**23**	26.936	1093.5734	961.5389 [M − H − 132]^−^, 637.4333 [M − H − 132 − 162 − 162]^−^, 475.3832 [M − H − 132 − 162 − 162 − 162]^−^, 391.2872	0.093	sanchirhinoside A_6_
**24**	28.314	1093.5719	961.5374 [M − H − 132]^−^, 637. 4382 [M − H − 132 − 162 − 162]^−^, 475.3753 [M − H − 132 − 162 − 162 − 162]^−^, 391.2883	0.064	isomer of sanchirhinoside A_6_ [19,28]
**25**	28.962	961.5374	799.4883 [M − H − 162]^−^, 637.4389 [M − H − 162 − 162]^−^, 475.3826 [M − H − 162 − 162 − 162]^−^, 391.2872	0.328	notoginsenoside R_3_
**26**	29.751	1093.5705	961.5272 [M − H − 132]^−^, 637.4367 [M − H − 132 − 162 − 162]^−^, 475.3753 [M − H − 132 − 162 − 162 − 162]^−^, 391.2879	0.028	isomer of sanchirhinoside A_6_ [19,28]
**27**	30.175	961.5305	799.4817 [M − H − 162]^−^, 637.4185 [M − H − 162 − 162]^−^, 475.3757 [M − H − 162 − 162 − 162]^−^, 391.2901	0.187	notoginsenoside N
**28**	30.175	1107.5870	961.5311 [M − H − 146]^−^, 799.4749 [M − H − 146 − 162]^−^, 637.4310 [M − H − 146 − 162 − 162]^−^, 475.3794 [M − H − 146 − 162 − 162 − 162]^−^, 391.2892	−	yesanchinoside E [17]
**29**	30.599	961.5303	637.4328 [M − H − 162 − 162]^−^, 475.3781 [M − H − 162 − 162 − 162]^−^, 391.2912	0.170	notoginsenoside R_6_
**30**	30.999	1107.5789	961.5299 [M − H − 146]^−^, 799.4747 [M − H − 146 − 162]^−^, 637.4224 [M − H − 146 − 162 − 162]^−^, 475.3760 [M − H − 146 − 162 − 162 − 162]^−^, 391.2902	0.026	isomer of yesanchinoside E [17]
**31**	31.788	961.5294	799.4784 [M − H − 162]^−^, 637.4286 [M − H − 162 − 162]^−^, 475.3782 [M − H − 162 − 162 − 162]^−^, 391.2899	0.715	20-*O-*glucoginsenoside Rf
**32**	32.259	961.5289	799.4824 [M − H − 162]^−^, 637.4403 [M − H − 162 − 162]^−^, 475.3832 [M − H − 162 − 162 − 162]^−^, 391.2876	0.052	isomer of 20-*O-*glucoginsenoside Rf
**33**	33.661	931.5210	637.4369 [M − H − 132 − 162]^−^, 475.3824 [M − H − 132 − 162 − 162]^−^,391.3270	13.39	notoginsenoside R_1_
**34**	34.296	961.5297	799.4803 [M − H − 162]^−^, 637.4268 [M − H − 162 − 162]^−^, 475.3785 [M − H − 162 − 162 − 162]^−^, 391.2913	0.151	notoginsenoside M or N [19,21]
**35**	34.991	961.5295	799.4811 [M − H − 162]^−^, 637.4194 [M − H − 162 − 162]^−^, 475.3634 [M − H − 162 − 162 − 162]^−^, 391.2920	0.067	isomer of notoginsenoside M or N [19,21]
**36**	35.392	931.5179	637.4281 [M − H − 132 − 162]^−^, 475.3762 [M − H − 132 − 162 − 162]^−^, 391.2901	0.138	gypenoside LXIV [29]
**37**	35.957	961.5277	799.4883 [M − H − 162]^−^, 637.4255 [M − H − 162 − 162]^−^, 475.3787 [M − H − 162 − 162 − 162]^−^, 391.2877	0.041	isomer of notoginsenoside R_3_ or isomer of notoginsenoside R_6_ [17,19]
**38**	36.31	1121.5639	959.3973 [M − H − 162]^−^, 797.4040 [M − H − 162 − 162]^−^, 473.3085 [M − H − 162 − 162 − 162 − 162]^−^	0.229	quinquenoside IV [27]
**39**	36.31	959.1231	797.3990 [M − H − 162]^−^, 635.4110 [M − H − 162 − 162]^−^, 473.3675 [M − H − 162 − 162 − 162]^−^	−	notoginsenoside G
**40**	36.781	799.4673	637.4352 [M − H − 162]^−^, 475.3815 [M − H − 162 − 162]^−^, 391.2930	27.59	ginsenoside Rg_1_ [27]
**41**	37.3	945.5324	783.4775 [M − H − 162]^−^, 637.4309 [M − H − 162 − 146]^−^, 475.3791 [M − H − 162 − 146 − 162]^−^, 391.3001	7.670	ginsenoside Re
**42**	37.676	1255.6201	1123.1027 [M − H − 132]^−^, 961.2952 [M − H − 132 − 162]^−^, 799.3770 [M − H − 132 − 162 − 162]^−^, 637.4266 [M − H − 132 − 162 − 162 − 162]^−^, 475.3701 [M − H − 132 − 162 − 162 − 162 − 162]^−^, 391.2900	0.032	PPT-A-S_1_(Glc-Glc-Xyl/Ara)-S_2_(Glc-Glc) [21]
**43**	39.349	1141.5875	817.4870 [M − H − 162 − 162]^−^, 655.4351 [M − H − 162 − 162 − 162]^−^, 493.3991 [M − H − 162 − 162 − 162 − 162]^−^	0.222	quinquenoside L_16_ [18]
**44**	39.349	979.5352	817.4912 [M − H − 162]^−^, 655.4429 [M − H − 162 − 162]^−^, 493.4046 [M − H − 162 − 162 − 162]^−^	−	PPT-C-S_1_(Glc)-S_2_(Glc-Glc) [30]
**45**	41.41	1123.5721	961.5210 [M − H − 162]^−^, 799.4757 [M − H − 162 − 162]^−^, 637.4238 [M − H − 162 − 162 − 162]^−^, 475.3770 [M − H − 162 − 162 − 162 − 162]^−^, 391.2870	0.248	PPT-A-S_1_(Glc-Glc)-S_2_(Glc-Glc) [31]
**46**	41.41	901.5177	637.4241 [M − H − 132 − 132]^−^, 475.3827 [M − H − 132 − 132 − 162]^−^, 391.2881	−	chikusetsusaponin L_5_ [27]
**47**	41.41	769.3977	637.3997 [M − H − 162]^−^, 475.3876 [M − H − 162 − 132]^−^, 391.2851	−	pseudoginsenoside Rt_3_
**48**	42.717	901.5008	637.4230 [M − H − 132 − 132]^−^, 475.3744 [M − H − 132 − 132 − 162]^−^, 391.2901	0.077	isomer of chikusetsusaponin L_5_ [27]
**49**	43.223	769.4713	637.4342 [M − H − 132]^−^, 475.3828 [M − H − 132 − 162]^−^, 391.2873	0.090	notoginsenoside R_2_ [19]
**50**	43.8	959.5032	797.4575 [M − H − 162]^−^, 635.4076 [M − H − 162 − 162]^−^, 473.3611 [M − H − 162 − 162 − 162]^−^	0.145	isomer of OCO-D-S_1_(Glc-Glc-Glc)*
**51**	44.377	1109.5496	785.4931 [M − H − 162 − 162]^−^, 623.4047 [M − H − 162 − 162 − 162]^−^, 491.3606 [M − H − 162 − 162 − 162 − 132]^−^, 391.8654	0.032	OCO-B-S_1_(Xyl-Glc-Glc-Glc)-S_2_(OH)*
**52**	44.79	769.4673	637.4342 [M − H − 132]^−^, 475.3828 [M − H − 132 − 162]^−^, 391.2890	0.045	isomer of notoginsenoside R_2_ [19]
**53**	45.108	961.5108	799.4836 [M − H − 162]^−^, 637.4239 [M − H − 162 − 162]^−^, 475.3783 [M − H − 162 − 162 − 162]^−^, 391.2903	0.174	isomer of 20-*O-*glucoginsenoside R_f_ [32,33]
**54**	45.108	1123.5610	961.5215 [M − H − 162]^−^, 799.4657 [M − H − 162 − 162]^−^, 637.4338 [M − H − 162 − 162 − 162]^−^, 475.3770 [M − H − 162 − 162 − 162 − 162]^−^, 391.2891	−	isomer of PPT-A-S_1_(Glc-Glc)-S_2_(Glc-Glc) [31]
**55**	46.344	769.4412	637.4341 [M − H − 132]^−^,475.3807 [M − H − 132 − 162]^−^,391.2891	0.196	sanchirhinoside A_3_ [19]
**56**	46.344	915.5043	783.4801 [M − H − 132]^−^, 637.4801 [M − H − 132 − 146]^−^, 475.3756 [M − H − 132 − 146 − 162]^−^, 391.3001	−	PPT-A-S_1_(Rha-Xyl)-S_2_(Glc) [31]
**57**	46.909	1123.5456	961.5244 [M − H − 162]^−^, 799.4729 [M − H − 162 − 162]^−^, 637.4283 [M − H − 162 − 162 − 162]^−^, 475.3721 [M − H − 162 − 162 − 162 − 162]^−^, 391.2911	0.051	isomer of PPT-A-S_1_(Glc-Glc)-S_2_(Glc-Glc) [31]
**58**	47.169	1123.5427	961.5227 [M − H − 162]^−^, 799.4758 [M − H − 162 − 162]^−^, 637.4274 [M − H − 162 − 162 − 162]^−^, 475.3728 [M − H − 162 − 162 − 162 − 162]^−^, 391.2893	0.012	isomer of PPT-A-S_1_(Glc-Glc)-S_2_(Glc-Glc) [31]
**59**	47.84	1121.5226	959.5057 [M − H − 162]^−^, 797.4549 [M − H − 162 − 162]^−^, 635.4088 [M − H − 162 − 162 − 162]^−^, 473.3567 [M − H − 162 − 162 − 162 − 162]^−^	0.077	OCO-D-S_1_(Glc-Glc-Glc-Glc) *
**60**	48.17	1125.5476	963.5411 [M − H − 162]^−^, 801.4900 [M − H − 162 − 162]^−^, 635.4389 [M − H − 162 − 162 − 162]^−^, 477.3889 [M − H − 162 − 162 − 162 − 162]^−^	0.066	PPD-B-S_1_(Glc-Glc)-S_2_(Glc-Glc) [34]
**61**	48.594	1123.5306	961.5171 [M − H − 162]^−^, 799.4745 [M − H − 162 − 162]^−^, 637.4232 [M − H − 162 − 162 − 162]^−^, 475.3754 [M − H − 162 − 162 − 162 − 162]^−^, 391.2891	0.035	isomer of PPT-A-S_1_(Glc-Glc)-S_2_(Glc-Glc) [31]
**62**	48.864	797.4781	635.4190 [M − H − 162]^−^, 473.3695 [M − H − 162 − 162]^−^	0.039	PPT-d-S_1_(Glc-Glc) [21]
**63**	49.63	1123.5254	961.5203 [M − H − 162]^−^, 799.4698 [M − H − 162 − 162]^−^, 637.4431 [M − H − 162 − 162 − 162]^−^, 475.3701 [M − H − 162 − 162 − 162 − 162]^−^, 391.2973	0.097	isomer of PPT-A-S_1_(Glc-Glc)-S_2_(Glc-Glc) [31]
**64**	49.63	947.4713	815.3567 [M − H − 132]^−^, 653.3778 [M − H − 132 − 162]^−^, 491.3608 [M − H − 132 − 162 − 162]^−^, 391.8554	−	OCO-B-S_1_(Glc-Glc-Xyl/Ara)-S_2_(OH) [35]
**65**	50.101	961.4723	799.4722 [M − H − 162]^−^, 637.4220 [M − H − 162 − 162]^−^, 475.3720 [M − H − 162 − 162 − 162]^−^	0.1160	vina−ginsenoside R_4_
**66**	50.254	781.4321	619.3533 [M − H − 162]^−^, 457.3661 [M − H − 162 − 162]^−^	0.032	sanchirhinoside B
**67**	50.619	961.4687	799.4598 [M − H − 162]^−^, 637.4037 [M − H − 162 − 162]^−^, 475.3770 [M − H − 162 − 162 − 162]^−^, 391.2866	0.058	isomer of 20-O*-*glucoginsenoside R_f_ [32,33]
**68**	51.762	961.4623	781.4714 [M − H − 180]^−^, 637.4232 [M − H − 180 − 144]^−^, 475.3801 [M − H − 180 − 144 − 162]^−^, 391.2909	0.019	isomer of notoginsenoside R_3_ or isomer of notoginsenoside R_6_ [17,19]
**69**	52.221	799.4215	637.4209 [M − H − 162]^−^, 475.3722 [M − H − 162 − 162]^−^, 391.2877	0.068	isomer of ginsenoside Rg_1_ [36,37]
**70**	53.552	961.4620	799.4434 [M − H − 162]^−^, 637.4263 [M − H − 162 − 162]^−^, 475.4024 [M − H − 162 − 162 − 162]^−^, 391.2907	0.039	isomer of 20-*O-*glucoginsenoside R_f_ [32,33]
**71**	54.376	961.4630	799.4695 [M − H − 162]^−^, 637.4074 [M − H − 162 − 162]^−^, 475.3638 [M − H − 162 − 162 − 162]^−^, 391.2881	0.019	isomer of notoginsenoside R_3_ or isomer of notoginsenoside R_6_ [17,19]
**72**	55.185	769.4795	637.4336 [M − H − 132]^−^, 475.3814 [M − H − 132 − 162]^−^, 391.2869	1.572	isomer of notoginsenoside R_2_
**73**	55.813	799.4331	637.4356 [M − H − 162]^−^, 475.4315 [M − H − 162 − 162]^−^, 391.2872	0.206	notoginsenoside U
**74**	56.461	959.4537	797.4608 [M − H − 162]^−^, 635.4138 [M − H − 162 − 162]^−^, 473.3590 [M − H − 162 − 162 − 162]^−^	0.026	ginsenoside III or vinaginsenoside R_20_ [38]
**75**	56.767	1371.5895	1107.5948 [M − H − 132 − 132]^−^, 945.5704 [M − H − 132 − 132 − 162]^−^, 783.4893 [M − H − 132 − 132 − 162 − 162]^−^, 459.5118 [M − H − 132 − 132 − 162 − 162 − 162 − 162]^−^	0.296	notoginsenoside D [17]
**76**	57.992	901.4601	769.4200 [M − H − 132]^−^, 637.4355 [M − H − 132 − 132]^−^, 475.4321 [M − H − 132 − 132 − 162]^−^	0.171	chikusetsusaponin L_5_ [39]
**77**	58.392	901.4614	769.4231 [M − H − 132]^−^, 637.4352 [M − H − 132 − 132]^−^, 475.4335 [M − H − 132 − 132 − 162]^−^	0.602	notoginsenoside Rw1 [21]
**78**	58.569	783.4448	621.3941 [M − H − 146]^−^, 475.3495 [M − H − 146 − 162]^−^, 391.2896	0.754	ginsenoside Rg_2_
**79**	59.146	1239.5732	1107.6034 [M − H − 132]^−^, 945.5396 [M − H − 132 − 162]^−^, 783.5018 [M − H − 132 − 162 − 162]^−^, 621.4381 [M − H − 132 − 162 − 162 − 162]^−^, 459.5110 [M − H − 132 − 162 − 162 − 162 − 162]^−^	2.087	chikusetsusaponinVI [40]
**80**	60.406	637.3796	475.3437 [M − H − 162]^−^,391.2901	0.103	ginsenoside F_1_ [27]
**81**	60.818	1269.5894	1107.5013 [M − H − 162]^−^, 945.4587 [M − H − 162 − 162]^−^, 783.4201 [M − H − 162 − 162 − 162]^−^, 621.3833 [M − H − 162 − 162 − 162 − 162]^−^, 459.5120 [M − H − 162 − 162 − 162 − 162 − 162]^−^	0.045	ginsenoside Ra_0_ or quinquenoside V [19,21]
**82**	61.572	1239.5892	1107.5974 [M − H − 132]^−^, 945.5267 [M − H − 132 − 162]^−^, 783.4965 [M − H − 132 − 162 − 162]^−^, 621.4475 [M − H − 132 − 162 − 162 − 162]^−^, 459.5121 [M − H − 132 − 162 − 162 − 162 − 162]^−^	2.622	notoginsenoside Fa
**83**	62.102	1105.5357	943.5543 [M − H − 162]^−^, 781.4996 [M − H − 162 − 162]^−^, 619.3441 [M − H − 162 − 162 − 162]^−^, 457.3654 [M − H − 162 − 162 − 162 − 162]^−^	0.941	PPT-B-S_1_(Glc-Glc-Glc-Glc) [38]
**84**	63.562	1105.5425	943.5521 [M − H − 162]^−^, 781.5010 [M − H − 162 − 162]^−^, 619.3501 [M − H − 162 − 162 − 162]^−^, 457.2601 [M − H − 162 − 162 − 162 − 162]^−^	0.161	isomer of PPT-B-S_1_(Glc-Glc-Glc-Glc) [38]
**85**	63.562	1239.2331	1077.4231 [M − H − 162]^−^, 915.4635 [M − H − 162 − 162]^−^, 621.3862 [M − H − 162 − 162 − 294]^−^, 459.3452 [M − H − 162 − 162 − 294 − 162]^−^	0.019	ginsenoside Ra_3_ [39]
**86**	64.198	1107.5642	945.4624 [M − H − 162]^−^, 783.4239 [M − H − 162 − 162]^−^, 621.3852 [M − H − 162 − 162 − 162]^−^, 459.3475 [M − H − 162 − 162 − 162 − 162]^−^	32.21	ginsenoside Rb_1_
**87**	66.483	1107.5632	945.4631 [M − H − 162]^−^, 783.4197 [M − H − 162 − 162]^−^, 621.3800 [M − H − 162 − 162 − 162]^−^, 459.3452 [M − H − 162 − 162 − 162 − 162]^−^	0.019	isomer of ginsenoside Rb_1_ [12,40]
**88**	68.791	1077.5696	945.4812 [M − H − 132]^−^, 783.4405 [M − H − 132 − 162]^−^, 621.4014 [M − H − 132 − 162 − 162]^−^, 459.3421 [M − H − 132 − 162 − 162 − 162]^−^	0.110	notoginsenoside L [12,27]
**89**	69.639	1077.5705	945.4888 [M − H − 132]^−^, 783.4465 [M − H − 132 − 162]^−^, 621.4008 [M − H − 132 − 162 − 162]^−^, 459.3544 [M − H − 132 − 162 − 162 − 162]^−^	0.080	isomer of notoginsenoside L [12,27]
**90**	70.487	943.5121	781.4300 [M − H − 162]^−^, 619.3881 [M − H − 162 − 162]^−^,457.3656 [M − H − 162 − 162 − 162]^−^	0.058	PPT-B-S_1_(Glc-Glc-Glc) *
**91**	73.384	945.5495	783.4954 [M − H − 162]^−^, 621.4422 [M − H − 162 − 162]^−^,459.3887 [M − H − 162 − 162 − 162]^−^	3.134	ginsenoside Rd
**92**	77.824	945.5520	783.4964 [M − H − 162]^−^, 621.4415 [M − H − 162 − 162]^−^,459.3887 [M − H − 162 − 162 − 162]^−^	0.370	isomer of ginsenoside Rd [9,12]
**93**	83.312	619.4279	457.3652 [M − H − 162]^−^	0.019	ginsenoside Rk_3_ [41,42]
**94**	85.608	619.4255	457.3686 [M − H − 162]^−^	0.067	ginsenoside Rh_4_ [41,42]

*: new compounds.

[M − H]^−^ (*m/z* 961.5409) and [M + COOH]^−^ (*m/z* 1007.5460) peaks of compound **25** were observed in the negative MS spectrum, and the peak ratio of [M − H]^−^ to [M + COOH]^−^ was 1.5, which indicated more than one sugar was located at the C-20 position. Diagnostic fragment ions at *m/z* 799.4883 [M − H − 162]^−^, 637.4389 [M − H − 162 − 162]^−^, 475.3826 [M − H − 162 − 162 − 162]^−^, 391.2872 as well as *m/z* 405.3973, 423.4204, 441.4223 were observed in negative and positive mode, respectively. As a result, compound **25** was identified as notoginsenoside R_3_, and compounds **37** and **71** were identified as isomers of **25** because similar fragment ions were observed in their spectra.

Compound **90** produced [M − H]^−^ ions at *m/z* 943.5121 and [M + COOH]^−^ ions at *m/z* 989.5183 in negative mode, while the peak ratio of [M − H]^−^ to [M + COOH]^−^ was 8, which indicated there are two or more sugars at the C-20 position. Diagnostic ions of PPT type were observed at *m/z* 405.3433, 423.3545, 441.3794, and diagnostic ions of PPT-A type were observed at *m/z* 457.3656. In the MS/MS spectrum, characteristic fragment ions at *m/z* 781.4300 [M − H − 162]^−^, 619.3881 [M − H − 162 − 162]^−^, 457.3656 [M − H − 162 − 162]^−^ and Glc-Glc ions at *m/z* 221.0662 were obtained. Based on the MS cleavage rules (Table 1 and Table 2), literature data and a SciFinder database search, compound **90** was tentatively identified as PPT-B-S_1_(Glc-Glc-Glc). 

The molecular weight of compound **83** could be supposed to 1106 through negative ions at *m/z* 1105.5357 [M − H]^—^ and 1151.5401 [M + COOH]^−^ (Table 1 and Table 2). There must be less than one sugar at C-20 position according to the peak ratio of [M − H]^−^ to [M + COOH]^−^(0.1). Furthermore, diagnostic ions of PPT type were observed at *m/z* 405.3459, 423.3565, 441.3661. In the MS/MS spectrum, fragment ions at *m/z* 943.5543 [M − H − 162]^−^, 781.4996 [M − H − 162 − 162]^−^, 619.3441 [M − H − 162 − 162 − 162]^−^, 457.3654 [M − H − 162 − 162 − 162 − 162]^−^ could be deduced to represent neutral losses of four glucose moieties successively from the precursor ions. Glc-Glc diagnostic ions at *m/z* 221.0664 could be detected. Characteristic ions at *m/z* 457 indicated the aglycone type of **83** should be PPT-B (Figure 1). According to the above elucidation and literature data, **83** could be tentatively identified as PPT-B-S_1_(Glc-Glc-Glc-Glc). Compound **84** with a similar fragmentation behavior could be tentatively identified as an isomer of **83** (Table 1 and Table 2). 

Compound **12** produced [M − H]^−^ ions at *m/z* 817.4872 and [M + COOH]^−^ ions at *m/z* 863.4982 in the negative spectrum, while the peak ratio of [M − H]^−^ to [M + COOH]^−^ was 0.8, which indicated there is more than one sugar at C-20. In MS/MS mode, characteristic fragment ions at *m/z* 655.4462 [M − H − 162]^−^, 493.3934 [M − H − 162 − 162]^−^ and 323. 2112 [Glc-Glc]^−^ were obtained. In positive mode, it produced ions at *m/z* 423.3736 and 441.3848, which could be used to identify it as a PPT type, and in MS/MS mode, PPT-C type characteristic fragment ions at *m/z* 493.3934 was observed. According to retention time and MS fragment rules, compound **12** was tentatively identified as PPT-C-S_1_(Glc-Glc)-S_2_(H). 

By utilizing MS cleavage rules, as well as comparison of the retention time to references, compounds **27**, **29**, **31**, **33**, **40**, **41**, **47**, **55**, **66**, **73**, **78**, **93** and **94** were unambiguously identified as notoginsenoside N, notoginsenoside R_6_, 20-*O-*glucoginsenoside Rf, notoginsenoside R_1_, ginsenoside Rg_1_,ginsenoside Re, pseudoginsenoside Rt_3_, sanchirhinoside A_3_, sanchirhinoside B, notoginsenoside U, ginsenoside Rg_2_, ginsenoside Rk_3_, and ginsenoside Rh_4_, respectively (Table 1 and Table 2).

Similarly, by utilizing MS cleavage rules and literature data, another 27 PPT type saponins were identified or tentatively identified as ginsenoside Rf (**13**), yesanchinoside E (**28**), an isomer of yesanchinoside E (**30**), an isomer of 20-*O-*glucoginsenoside Rf (**32**), notoginsenoside M or N (**34**), an isomer of notoginsenoside M or N (**35**), gypenoside LXIV (**36**), an isomer of notoginsenoside R_3_ or notoginsenoside R_6_ (**37**), PPT-A-S_1_(Glc-Glc-Xyl/Ara)-S_2_(Glc-Glc) (**42**), quinquenoside L_16_ (**43)**, PPT-C-S_1_(Glc)-S_2_(Glc-Glc) (**44)**, PPT-A-S_1_(Glc-Glc)-S_2_(Glc-Glc) (**45**), chikusetsusaponin L_5_ (**46**), an isomer of chikusetsusaponin L_5_ (**48**), an isomer of notoginsenoside R_2_ (**49**), an isomer of notoginsenoside R_2_ (**52**), an isomer of 20-*O*-glucoginsenoside Rf (**53**), an isomer of PPT-A-S_1_(Glc-Glc)-S_2_(Glc-Glc) (**54**), PPT-A-S_1_(Rha-Xyl)-S_2_(Glc) (**56**), an isomer of PPT-A-S_1_(Glc-Glc)-S_2_(Glc-Glc) (57), an isomer of PPT-A-S_1_(Glc-Glc)-S_2_(Glc-Glc) (**58**), an isomer of PPT-A-S_1_(Glc-Glc)-S_2_(Glc-Glc) (**61**), an isomer of PPT-A-S_1_(Glc-Glc)-S_2_(Glc-Glc) (**63**), an isomer of 20-*O*-glucoginsenoside Rf (**67**), an isomer of notoginsenoside R_3_ or an isomer of notoginsenoside R_6_ (**68**), ginsenoside Rg_1_ (**69**), an isomer of 20-O*-*glucoginsenoside Rf (**70**), an isomer of notoginsenoside R_3_ or an isomer of notoginsenoside R_6_ (**71**), an isomer of notoginsenoside R_2_(**72**) and ginsenoside F_1_ (**78**), respectively (Table 1 and Table 2).

#### 2.3.3. Identification of OCO Type Saponins

The molecular weight of compound **9** could be supposed to 816 through negative ions at *m/z* 861.4817 [M + COOH]^−^ and 815.4669 [M − H]^−^ (Figure 5A,B). There must be less than one sugar moiety at the C-20 position according to the peak ratio of [M − H]^−^ to [M + COOH]^−^(0.02). Furthermore, diagnostic ions of OCO type was observed at *m/z* 421, 439, 457 (Figure 1 and Figure 5B). In the MS/MS spectrum of [M + HCOO]^−^ (Figure 5C), fragment ions at *m/z* 653.4263, 491.3777 could be deduced to correspond to neutral losses of two glucoses successively from the precursor ions. Diagnostic ions at *m/z* 323 could be used for confirming a Glc-Glc chain located at the C-6 position. Characteristic ions at *m/z* 415 and 491 indicated the aglycone type of **9** should be OCO-C (Figure 1 and Figure 5C). According to the above elucidation and literature data, **9** could be tentatively identified as majonoside R_1_ [26,43]. Compounds **10**, **11**, **16**, **17** had similar fragmentation behaviors at *m/z* 653.4263 [M − H − 162]^−^, 491.3777 [M − H − 162 − 162]^−^, 415.3253. Glc-Glc diagnostic ions at *m/z* 323 and diagnostic ions of OCO type was also observed at *m/z* 421, 439, 457, so compounds **10**, **11**, **16**, **17** were tentatively identified as isomers of majonoside R_1_ since similar characteristic fragment ions were obtained (Table 1 and Table 2).

[M − H]^−^ (*m/z* 947.4713) and [M + COOH]^−^ (*m/z* 993.4633) of compound **64** were observed in the negative MS spectrum, and the peak ratio of [M − H]^−^ to [M + COOH]^−^ was 0.25. Diagnostic fragment ions at *m/z* 815.3567 [M − H − 132]^−^, 653.3778 [M − H − 132 − 162]^−^, 491.3608 [M − H − 132 − 162 − 162]^−^, 391.8554, as well as *m/z* 421.3447, 439.3612, 457.3701 were observed in negative and positive mode, respectively. As a result, **64** was identified as OCO-B-S_1_(Glc-Glc-Xyl/Ara)-S_2_(OH).

The molecular weight of compound **1** could be deduced as 966 according to quasi−molecular ions at *m/z* 965.5198 [M − H]^−^ and 1011.5278 [M + COOH]^−^. The peak ratio of [M − H]^−^ to [M + COOH]^−^ was about 0.1, which indicated that there was one or less sugar at C-20. The aglycone type could be identified as OCO type through diagnostic ions at *m/z* 439, 457. In the MS/MS spectrum of [M − H]^−^, fragment ions at *m/z* 785.9465 [M − H − 180]^−^, 653.3969 [M − H − 180 − 132]^−^, 491.3843 [M − H − 180 − 132 − 162]^−^, 415.3350 and fragment ions at *m/z* 191 indicated there was a Glc-Xyl/Ara chain in compound **1**. Characteristic ions at *m/z* 415 and 491 indicated the aglycone would be OCO-A type. According to the peak ratio rule of [M − H]^−^ to [M + COOH]^−^ and the structural features of OCO-A, we could deduce that the Glc-Xyl chain was linked at the C-6 position of the aglycone. As a result, compound **1** was tentatively identified as OCO-A-S_1_(Glc)-S_2_(Glc-Xyl/Ara), compound **2** was identified as an isomer of **1** because a similar MS cleavage pathway was observed. 

**Figure 6:**
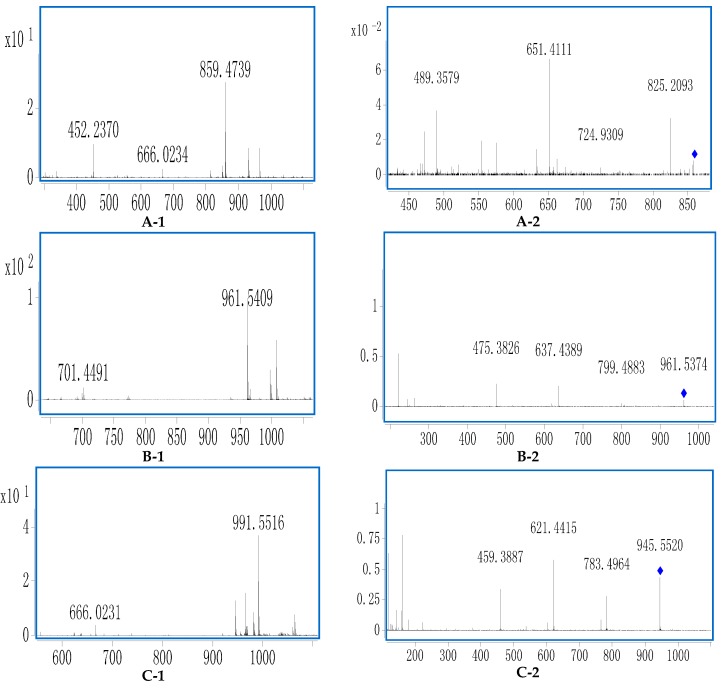
The typical TOF-MS spectra of compounds **18** (**A**); **25** (**B**); **92** (**C**) in negative mode (1: MS spectra; 2: MS/MS spectra).

Compound **18** could be supposed to have a molecular weight of 814 through negative ions at *m/z* 813.4677 [M − H]^−^ and 859.4739 [M + COOH]^−^ (Figure 6). There must be less than one sugar at the C-20 position according to the peak ratio (0.1) of [M − H]^−^ to [M + COOH]^−^. Furthermore, diagnostic ions of OCO type was observed at *m/z* 421, 439, 457 (Figure 1). In the MS/MS spectrum, fragment ions at *m/z* 651.4111 [M − H − 162]^−^ and 489.3579 [M − H − 162 − 162]^−^ could be deduced as successive neutral losses of two glucose molecules (Figure 6). Characteristic ions at *m/z* 489 indicated the aglycone type should be OCO-E. According to the above elucidation and literature data, **18** could be tentatively identified as OCO-E-S_1_(Glc-Glc). Compound **21** with similar fragmentation behaviors at *m/z* 651.4113 [M − H − 162]^−^, 489.3635 [M − H − 162 − 162]^−^, was tentatively identified as an isomer of OCO-E-S_1_(Glc-Glc).

In the negative MS spectrum of compound **20**, [M − H]^−^ ions at *m/z* 959.5194 and [M + COOH]^−^ at 1005.5220 as well as their peak ratio (0.25) were observed. In the MS/MS spectrum, characteristic fragment ions at *m/z* 797.4207 [M − H − 162]^−^, 635.3567 [M − H − 162 − 162]^−^, 473.3460 [M − H − 162 − 162 − 162]^−^ and 221.0431 [Glc-Glc]^−^ were also observed. In the positive spectrum it produced ions at *m/z* 421, 439, 457 which could be used to identify an OCO type compound. In MS/MS mode, OCO-D type fragment ions at *m/z* 473 could be obtained. According to the MS fragment rules, **20** was tentatively identified as OCO-D-S_1_(Glc-Glc-Glc). Similar diagnostic fragment ions were observed in **22** (Table 1 and Table 2).

Similarly, with the help of MS cleavage rules and literature values, compounds **7**, **14**, **15**, **19**, **51** were tentatively identified as vinaginsenoside R_5_ or yesanchinoside C, OCO-B-S_1_(Glc-Rha-Glc)-S_2_(CH_3_), an isomer of OCO-B-S_1_(Glc-Rha-Glc)-S_2_(CH_3_), an isomer of OCO-B-S_1_(Glc-Rha-Glc)-S_2_(CH_3_) and OCO-B-S_1_(Xyl-Glc-Glc-Glc)-S_2_(OH) (Table 1 and Table 2).

## 3. Experimental Section

### 3.1. Reagents and Materials 

HPLC grade acetonitrile (ACN) and formic acid were purchased from Merck Technologies Inc. (Darmstadt, Germany), and Tedia Company Inc. (Fairfield, OH, USA). Deionized water was obtained from a Millipore Milli-Q water system (Bedford, MA, USA). All other reagents were of analytical purity. ZSYXST sample was obtained from Guangxi Wuzhou Pharmaceutical (Group) Co., Ltd. (Wuzhou, China). Twenty-one reference compounds: sanchirhinoside A_6_ (**23**), notoginsenoside R_3_ (**25**), notoginsenoside N (**27**), notoginsenoside R_6_ (**29**), 20(*S*)-20-*O*-glucoginsenoside Rf (**31**), notoginsenoside R_1_ (**33**), notoginsenoside G (**39**), ginsenoside Rg_1_ (**40**), ginsenoside Re (**41**), pseudoginsenoside Rt_3_ (**47**), sanchirhinoside A_3_ (**55**), vina-ginsenoside R_4_ (**65**), sanchirhinoside B (**66**), notoginsenoside R_2_ (**72**), notoginsenoside U (**73**), ginsenoside Rg_2_ (**78**), notoginsenoside Fa (**82**), ginsenoside Ra_3_ (**85**), ginsenoside Rb_1_ (**86**), ginsenoside Rd (**91**), ginsenoside Rk_3_ (**93**), and ginsenoside Rh_4_ (**94**) were isolated from ZSYXST by the authors. Their structures were elucidated by 1D and 2D NMR spectra [28,44].

### 3.2. Sample Preparation and PHPLC Chromatography Conditions

ZSYXST (10 mg)was dissolved in 19% acetonitrile (1 mL)to obtain sample 1, which was then centrifuged for 10 min at 14,000 rpm, and the supernatant of sample 1 was applied to a Shimadzu LC-8A PHPLC system (Shimadzu, Kyoto, Japan), equipped with a binary pump, an UV detector and a fraction collector(FRC-10A). Chromatographic separation was achieved on a Cosmosil-5C_18_ column (20 × 250 mm, 5 µm), (NACALAI, Kyoto, Japan). The mobile phase consisted of water (A) and ACN (B), using 19%–20% B at 0–25 min, 20%–30% B at 25–35 min, 30%–35% B at 35–45 min, 35%–45% B at 45–70 min, 45%–90% B at 70–75 min, 90% B at 75–76 min, 90%–19% B at 76–80 min, the flow rate was 8 mL/min.

**Figure 7:**
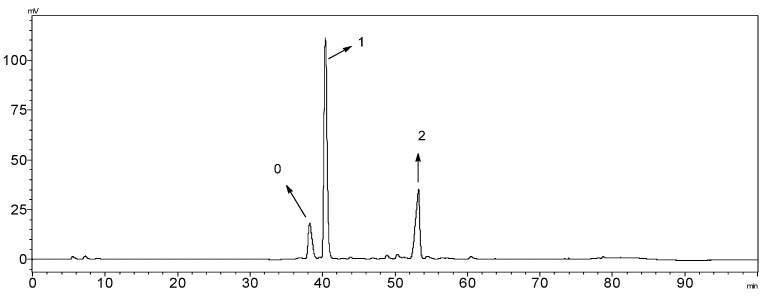
The PHPLC chromatogram of sample **1** (0: Notoginsenoside R_1_; 1: Ginsenoside Rg1 and Re; 2: Ginsenoside Rb1).

**Figure 8:**
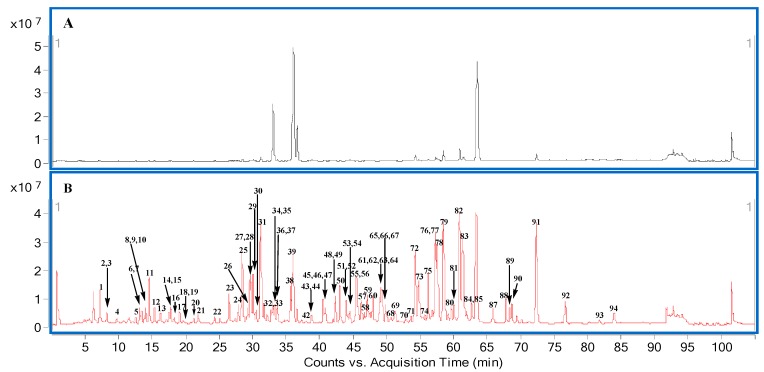
The total ion chromatograms of UHPLC-Q-TOF/MS in negative mode.

Utilizing an online collector simulation system, the major peaks 0–2 of ZSYXST were easily removed through the control of the collection parameters, set at level of 500 µV, peak slope of 1000 µV/s and the delay volume of 200 µL. Finally, sample 2 (1.3 mg) without major components was obtained, and the peaks of lower content ingredients became more obvious finally (Figure 7 and Figure 8).

### 3.3. UHPLC-Q-TOF/MS Conditions

Sample 2 (1 mg/mL) and ZSYXST sample (1 mg/mL) were analyzed on an Agilent 1290 Ultra high performance liquid chromatography system (Agilent, Palo alto, CA, USA), equipped with a T_3_ column (2.1 × 100 mm, 1.8 µm, Waters, Milford, MA, USA) under 40 °C. The mobile phase consisted of 0.1% formic acid water (A) and ACN (B), using 10% B at 0–10 min, 10−40% B at 10–90 min, 40%–100% B at 90–91 min, 100% B at 91–100 min, 100%–10% B at 100–100.1 min, 10% B at 100.1–105 min. The flow rate was 0.3 mL/min, and the sample volume injected was 2 µL.

The Q-TOF/MS analysis was performed on an Agilent 6520 Accurate-Mass Q-TOF/MS system. The conditions of the ESI source were: drying gas (N_2_) flow rate, 8.0 L/min; drying gas temperature, 350 °C; nebulizer, 30 psig; capillary voltage (Vcap), 3500V. Cone voltage was 120 V and 175 V in the positive and negative mode, respectively. Collision energy (CE) was dynamically adjusted from 45 to 70 V according to the *m/z* of precursor ions in the negative MS/MS mode. The mass analyzer scanned over *m/z* 100–2000. All the data were recorded and processed by Agilent Mass Hunter Workstation software (Version B.02.00).

## 4. Conclusions

In this work, a novel UHPLC-Q-TOF-MS combined with PHPLC method was established. The sensitivity of some minor components in ZSYXST could be enhanced significantly by this method. Combining the characteristic ions in positive and negative mode, the types of aglycone, saccharide, as well as the linkage positions of the saccharide chains of saponins were quickly determined. As a result, based on the exact mass, fragmentation behaviors, retention times and literature, 94 saponins, including 20 pairs of isomers, which could represent over 98% of the components in ZSYXST were identified or tentatively identified and ten of these saponins were identified as new compounds. This method could provide a powerful platform for profiling the compounds in ZSYXST and also could be useful for identification of saponins of *P. notoginseng* and *P. ginseng*.
